# Characterization of cell-type specific knockout of different elements of the endocannabinoid system in cortical glutamatergic neurons in the context of stress-induced behavioral phenotype

**DOI:** 10.1186/s42238-025-00368-7

**Published:** 2025-11-27

**Authors:** Margarita Tevosian, Alex F. Brown, Christina Schneider, Andrea Conrad, Ermelinda Lomazzo, Beat Lutz

**Affiliations:** 1 Institute of Physiological Chemistry, University Medical Center of the Johannes Gutenberg University, Mainz, Germany; 2 Leibniz Institute for Resilience Research (LIR), Mainz, Germany; 3 UHasselt, Dynamic Bioimaging Lab, Advanced Optical Microscopy Centre, Biomedical Research Institute, Agoralaan C (BIOMED), Diepenbeek, B3590 Belgium

**Keywords:** Stress, Chronic social defeat, Endocannabinoids, Anandamide, Behavior characterization

## Abstract

**Background:**

Chronic stress is an important factor for the development of mental health impairments, such as depression and generalized anxiety disorder. Chronic social defeat (CSD) stress is an ethologically valid model of chronic stress in rodents, combining elements of psychological and physical stress. The endocannabinoid (eCB) system plays important roles in maintaining the homeostasis of biological systems through the tuning of neuronal excitability, thereby mediating a protective role after prolonged stress exposure.

**Methods:**

In the present study, we investigated genetically modified adult male mice where the eCB signal via anandamide (AEA) was reduced (by deletion of the AEA synthesizing enzyme NAPE-PLD) or enhanced (by deletion of the AEA degradation enzyme FAAH), as well as mice lacking the cannabinoid CB1 receptor. These genetic manipulations were induced in glutamatergic neurons of the dorsal telencephalon. After the application of CSD stress, the phenotypes of these mutant mice were investigated in a battery of behavioral tests assessing sociability, anxiety, memory, shelter-seeking behavior, and despair.

**Results:**

We could confirm a robust anxiogenic effect of CSD in the EPM test. Interestingly, we have not observed a stress effect on the sociability of any of the mouse lines as identified in the SI test. Under non-stress conditions, we observed an anxiogenic phenotype in Glu-CB1-KO and Nex-NAPE-PLD KO, and hyperlocomotion in Nex-FAAH KO mice. Additionally, we could confirm a drastic reduction of FAAH protein levels in cortical and subcortical regions of Nex-FAAH line, and a moderate reduction of NAPE-PLD protein in cortical regions of Nex-NAPE-PLD KO mice.

**Conclusions:**

In conclusion, genetic manipulation of the endocannabinoid system in cortical glutamatergic neurons did not result in persistent effects of prolonged stress exposure. Detected differences between the genotypes in the non-stressed groups points toward baseline differences that could mask or over-power the effect of stress.

**Supplementary Information:**

The online version contains supplementary material available at 10.1186/s42238-025-00368-7.

## Background

Prolonged exposure to an aversive environment causes chronic stress, which exhausts the resources of an organism and often leads to the development of pathological states such as generalized anxiety disorder, depression, and others. The chronic social defeat (CSD) serves as a common chronic stress paradigm in mice. CSD follows construct, face, and predictive validity and has high translational and ethological value (Gray et al. 2015). Much work has been invested in characterizing this model and generating a detailed protocol, leading to a robust outcome of CSD: a depression-like phenotype with prominent anxiety, anhedonia, and social avoidance behaviors (Golden and HE, Berton O, Russo SJ. 2011).

The eCB system is thought to be a "buffering" component, protecting against detrimental effects of stress (Lutz et al. 2015). It primarily consists of the cannabinoid type 1 (CB1) and the type 2 (CB2) receptor, their lipid ligands 2-arachidonoyl glycerol (2-AG) and anandamide (N-arachidonoylethanolamide, AEA), and the synthesizing and degrading enzymes of eCBs. CB1 is abundantly expressed in the central nervous system (CNS): in GABAergic interneurons, glutamatergic, serotonergic, and noradrenergic neurons, and astrocytes (Lau and Schloss 2008; Cruz et al. 2016).

Studies on CB1 conditional knockout (KO) mice, such as the Glu-CB1-KO and GABA-CB1-KO lines, where CB1 is absent in cortical glutamatergic and in forebrain GABAergic neurons, respectively, revealed crucial involvement of the eCB system in anxiety behavior and stress processing (Rey et al. 2012; Bath et al. 2017; Dubreucq et al. 2012). Therefore, the presence of the CB1 in both glutamatergic and GABAergic neurons allows fine-tuning excitation and inhibition in the CNS.

The principal enzyme involved in the biosynthesis of AEA is N-acyl phosphatidylethanolamine phospholipase D (NAPE-PLD) (Schmid et al. 1990; Rahman et al. 2014). NAPE-PLD^−/−^ mice have been previously reported by three groups (Leung et al. 2006; Tsuboi et al. 2011; Leishman et al. 2016). Interestingly, the AEA levels were not reduced in the NAPE-PLD^−/−^ mouse line generated by the Cravatt laboratory, but were significantly reduced in two other lines generated by Luquet's and Deutsch's groups, the latter used in this study (Tsuboi et al. 2011).

FAAH is the primary enzyme responsible for the deactivation of AEA and has been extensively described and studied (Cravatt et al. 2001; McKinney and Cravatt 2005). Rapid termination of AEA activity occurs via the removal of AEA from the extracellular space and its hydrolysis to arachidonic acid (AA) and ethanolamine (Ahn et al. 2008). Previously described FAAH^−/−^ mouse line (Cravatt et al. 2001) displayed a 15-fold elevation of AEA levels and exhibited an array of enhanced CB1-mediated behaviors, such as catalepsy, hypothermia and others. Consequently, this line is not suitable for behavior characterization. Therefore, a newly-generated conditional FAAH KO mouse line was used in this study.

To evaluate the role of the eCB system in the processing of stress, we employed transgenic mouse lines targeting inactivation of the CB1 receptor, NAPE-PLD, and FAAH genes, respectively, specifically in cortical glutamatergic neurons. These mutant mice, together with wild-type controls, were exposed to the CSD stress paradigm, and their phenotypes were assessed using a battery of behavioral tests, estimating stress coping, anxiety, and general well-being.

## Materials and methods

### Animals

All experiments were performed according to the European Community's Council Directive of 22 September 2010 (2010/63EU) and approved by the respective agency of the State Rhineland-Palatinate (Landesuntersuchungsamt), registration number G-17–1–005. Male mice were group-housed in temperature- and humidity-controlled rooms with a 12-h light-dark cycle with water and food provided *ad libitum*. Animals were single-housed 7 days prior to behavioral experiments. Mice used in this study were 8 weeks old by the start of the experiments. Knockout animals (KO) were compared to appropriate wild-type littermate controls (WT), lacking the Cre transgene but containing both floxed alleles of the respective targeted gene. Genotyping was performed before and after experiments.

The Nex-Cre mouse line expresses Cre recombinase under the regulatory elements of *NEX*, which belongs to a family of helix-loop-helix (bHLH) proteins (Schwab et al. 2000). To achieve Cre expression, the coding region of *NEX* gene is substituted (on exon 2) by a Cre expression cassette. Mice in this line are therefore heterozygous for Nex-Cre. Cre expression is observed predominantly in dorsal telencephalic glutamatergic neurons (Goebbels et al. 2006).

In CB1 floxed mice, the mouse *CB1* gene is flanked by two loxP sites that are recognized by Cre recombinase (Marsicano et al. 2003). Glu-CB1-KO, were generated by crossing Nex-Cre and CB1-floxed mice (Monory et al. 2006). These animals lack CB1 in dorsal telencephalic glutamatergic neurons.

In NAPE-PLD floxed mice, exon 3 of the *NAPE-PLD* gene is flanked by two loxP sites, enabling Cre-mediated excision of exon 3 and subsequent inactivation of NAPE-PLD activity (Tsuboi et al. 2011). Nex^tg/wt^;NAPE-PLD^fl/fl^ mice were generated by crossing Nex-Cre mice with NAPE-PLD floxed mice, resulting in the knockout of *NAPE-PLD* in telencephalic glutamatergic neurons.

**Fig. 1:**
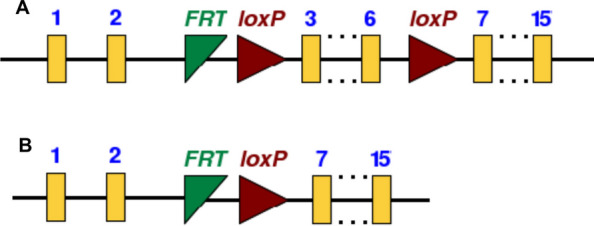
Schematic representation of the FAAH floxed line. **A** Exons 3 to 6 marked with flanked loxP sites. **B** Excision of exons 3 to 6 upon Cre activity. In yellow – exons 1 to 15. In red – loxP sites, recognized by the Cre. In green – flippase recognition target sites from selection steps in the generation of the modified gene locus

The FAAH floxed line was generated by genOway (France). Exons 3 to 6 of the *FAAH* gene were flanked by loxP sites, which are excised upon Cre activation (Fig. 1). The Cre-mediated deletion results in out-of-frame splicing and loss of all active sites and substrate binding domains (deletion of the N-terminal part of the amidase domain). A premature STOP codon in exon 7 appears when the out-of-frame splicing occurs. A truncated protein consisting of 143 amino acids will be expressed and contain one transmembrane helix without the predicted domain. A PCR was set up to enable the discrimination between animals with WT and conditional KO allele; the primers are indicated in Table 1. In a 50 µl reaction containing 30 ng gDNA and 15 pmol of primers, the PCR was performed with an initial denaturation at 94 °C (120 s), followed by denaturation at 94 °C (30 s), annealing at 65 °C (30 s) and extension at 68 °C (60 s) for 30 cycles, ending with a completion step at 68 °C for 480 s. The PCR yielded a product of either 178 bp (WT allele), or 261 bp (KO allele). A PCR product of 943 bp indicates a constitutive KO of *FAAH*. Importantly, after the deletion of exons 3–6, the truncated mRNA did not contain the protein sequence encoding the catalytic domain of the enzyme. Nex^tg/wt^;FAAH^fl/fl^ mice were generated by crossing Nex-Cre mice with FAAH-floxed mice, resulting in a knockout of *FAAH* in cortical glutamatergic neurons.

**Table 1:** List of primers used for genotyping. Bp – base pair

Line	Primer sequence 5' → 3'	Product bands
Nex-Cre	TCT TTT TCA TGT GCT CTT GG	390 bp – transgene allele
	CGC GCC TGA AGA TAT AGA AGA	
CB1^fl/fl^	GCT GTC TCT GGT CCT CTT AAA	500 bp – floxed allele 400 bp – WT allele 600 bp – germline deletion
	GGT GTC ACC TCT GAA AAC AGA	
	CTC CTG TAT GCC ATA GCT CTT	
NAPE-PLD^fl/fl^	CAC ACC CCA GGA GGC ATC ACA CT	538 bp – floxed allele 476 bp – WT allele 299 bp – germline deletion
	GAT GAG CTC GTC CAT TTC CAC CAT	
	GGT CTA TCT GTC GTT AGT GGC A	
FAAH^fl/fl^	AGAGTCCCAAGGAAAGGGGAGGATC	178 bp – WT allele 261 bp – floxed allele 943 bp – germline deletion
	AAGTCTAGGAGGCTCCAAACGCAGG	
	CAGGCAGATAGATAGACAGAAGGCTGTCAG	

### List of used primers

Genotyping before and after experiments was performed using genomic DNA from a tail biopsy. DNA isolation was performed using a standard method of proteinase K tissue lysis and subsequent isopropanol/ethanol DNA extraction. A PCR reaction was performed using primers specified below in Table 1.

### Chronic social defeat

CSD stress was carried out as previously reported (Krishnan et al. 2007; Tevosian et al. 2023) with minor modifications. Briefly, experimental mice and adult retired breeders CD1 mice (Charles River) were housed in the same cage but were physically separated by a perforated metal grid for 14 days. During this period, the separating grid was removed every day for 2 min per day to allow mouse interaction and the occurrence of multiple defeat episodes of attack from CD1 mice. After 14 days of CSD, mice were allowed to rest for one week (no exposure to CD1, single housing in the home cage) before starting the behavioral tests. Non-stressed animals were single-housed and handled daily. Mice were allocated to either the stress or control group in a pseudo-randomized fashion, matched according to their body weight.

### Behavioral assays

Behavioral tests were carried out during the light phase, and trials were video-recorded and analyzed with Noldus Ethovision XT software (Noldus, Wageningen, Netherlands). After each trial, the setups were cleaned with water.

### Social interaction

The social interaction test (SI) was performed as previously described (Krishnan et al. 2007; Tevosian et al. 2023). Briefly, experimental animals were placed into an open field box (40 × 27 × 40 cm) with a small circular enclosure at one wall of the box and allowed to explore the arena for 2.5 min and before being placed back to their home-cage. After introducing the social target – naïve mouse of CD1 strain (target) – into the enclosure, the experimental animal was re-introduced to the box and allowed to explore for another 2.5 min. Time in the interaction zone, defined as a circular zone 2.5 cm in diameter around the enclosure, was recorded automatically with tracking software (Noldus Ethovision XT).

The time the experimental animal spent in the interaction zone when the target mouse was absent (enclosure empty) was compared to time spent in the interaction zone when the target mouse was present. SI ratio was calculated as 100 x (time in the interaction zone with a target mouse present)/(time in the interaction zone with target absent).

### Light–dark test

The light–dark test (LDT) was carried out as previously reported (Tevosian et al. 2023; Guggenhuber et al. 2015; Ruehle et al. 2013) and used to test anxiety-like behavior. Briefly, experimental animals were placed into a custom box (39 × 39 cm), divided into the light zone (two thirds of the box, white walls) and dark zone (one third of the box, separated and covered by a 26 cm high lid, black walls) The light and dark compartments were connected with small entry zone (5 × 5 cm). Animals were allowed to explore freely and move between the dark and light zones for 6 min. Time spent in the light zone was assessed using video recording and subsequent automated analysis.

### Elevated plus maze

The elevated plus maze (EPM) was performed as previously reported (Tevosian et al. 2023; Ruehle et al. 2013) using a custom-made cross-shaped set-up having two open and two closed arms elevated 100 cm above the floor. The arms of the maze were 35 cm long and 6 cm wide. Experimental mice were placed into the maze facing the closed arms and were allowed to freely explore for 10 min. Animals were video-recorded and tracked automatically.

### Novel object habituation

The novel object habituation (NOH) represents a slight modification of the commonly used novel object recognition (NOR) test. Similarly to NOR, NOH tests preference for novelty in mice; introducing a 1 h delay between the two trials allows a better-defined and reproducible recognition of the novel object (data unpublished). Briefly, mice were allowed to explore an open white box (40 × 27 × 40 cm) (empty box, habituation trial, not scored) for 5 min and then again, the box containing an attractive and colorful object (object box, test trial-t1, scored) for 5 min. Then, 1 h later both habituation and test phase were performed again (object box, test trial-t2, scored) for 5 min. Data are expressed as percentage of exploration inhibition (EI) calculated as EI = 100-(t2/t1*100), where t corresponds to the time animals spent exploring the object in the initial test trial (t1) and the later test trial (t2). The positive values represent decreased interaction with the familiar object in the second test trial.

### Tail suspension test

The tail suspension test (TST) to measure emotional despair or depressive-like behavior followed the originally reported procedure with modifications (Steru et al. 1985). Mice were fixated by the tail onto a bright illuminated screen with fixative tape and left hanging for 6 min. The experimental animal was visually isolated from the nearest objects and recorded using a video-camera. Immobility was assessed automatically using tracking software.

### Nesting

Nesting behavioral test was performed and scored according to a previously established procedure (Deacon 2006) and was used to evaluate global well-being in mice and monitor whether CSD affects routinely performed tasks and shelter-seeking behavior. Briefly, a pad of compressed cotton was placed overnight into the home cage of the mouse. The following morning the quality and complexity of the nest were evaluated. A combined score (1 – no nest, 5 – perfect nest) was assigned for each mouse, combining the complexity of nest building and the amount of cotton that was left not processed.

### Preparation of tissue lysate

For dissection, the frozen brains were thawed in a dish of cold PBS for 3–5 min. The desired brain regions were then dissected (hippocampus (HC), striatum (STR), cortex (CX), thalamus (TH)), and transferred directly into a sample tube with RIPA Buffer and homogenized using a hand-held electric homogenizer (10–20 s depending on tissue size) and incubated for one hour at 4 °C with rotation.

After incubation, the samples were centrifuged at 4 °C at full speed for 10 min and the supernatant was transferred to a new sample tube and stored at -80 °C.

For sample preparation, the lysates were thawed on ice and the total protein concentration was determined using the Bradford assay.

### Western blot

From the tissue lysate, 5 μg of total protein from each sample was loaded into 10% acrylamide gels for SDS-PAGE. 10 μl of colour protein standard (NEB, P7719) protein ladder was also loaded onto each gel. The proteins from the gel were transferred to a nitrocellulose membrane over one hour. The membranes were stained with 0.1% Ponceau-S stain to validate the protein transfer. The stain was washed away and the membranes were cut with a sharp scalpel between the 34 and 43 kDa makers and just below the 55 kDa marker. The lowest section of the gel was blocked in 5% BSA in TBS-T (0.1% Tween-20) for 1 h at room temperature with gentle rocking, and then overnight at 4 °C with fresh BSA containing the anti-GAPDH antibody (Abcam; ab9484) diluted 1:1000. The middle section was blocked in 5% milk in TBS-T and then with anti-NAPE-PLD antibody (Frontier Institute/Nittobo Medical; Af720) in fresh blocking buffer diluted 1:250 overnight at 4 °C. The upper section was blocked in 5% milk and then incubated with anti-FAAH antibody (Abcam; ab54615) 1:2500 overnight at 4 °C. After washing the blots were incubated for 1 h at room temperature with the appropriate secondary antibody HRP conjugate (Jackson Immuno Research/Dianova) diluted 1:5000 in 5% milk.

The three sections to each blot were incubated together for 5 min with the ECL Prime Western blotting reagent (GE Healthcare Life Science, Uppsala, Sweden) in a dimly lit room. The blotting reagent was poured off and the blot sections were imaged using the UVP ChemStudio (analytic Jena, Jena, Germany) imager and analyzed with the VisionWorks 9.1 software. The chemiluminescence images were subjected to 1D analysis as directed by the software using 50% lane sensitivity and maximum lane sensitivity. Additional lanes were selected manually where required. The output of the analysis included the Intensity Volume (I-Vol) of each band. The I-Vol of the bands of interest (FAAH and NAPE-PLD) was divided by the I-Vol of the endogenous control band (GAPDH) within the same sample to generate the relative intensity; these values were plotted for each band of interest between genotypes and brain regions and analyzed with multiple unpaired t-tests with Holm-Šídák method for multiple comparison correction using GraphPad Prism 9.4.1.

### Data analysis

Data are represented as the mean ± standard error of the mean (SEM). Statistical analysis was performed using GraphPad Prism 6.0 (GraphPad Software, La Jolla, CA, USA). D'Agostino-Pearson normality test was performed for all groups. Unpaired two-tailed Student's t-test was used to analyze normally distributed data. Mann–Whitney test was performed in case of not normally distributed data. One or two-way analysis of variance (ANOVA) with Tukey's multiple comparison test was performed where applicable. *p* < 0.05 was set as a value to determine statistical significance. All reported p-values are two-tailed.

## Results

### Effects of conditional knockout of the CB1 receptor in cortical glutamatergic neurons on behavior after CSD stress

Glu-CB1-KO mice are lacking CB1 on glutamatergic neurons of the dorsal telencephalon. After subjecting the mice to CSD, we observed changes in their behavior (summarized in Supplementary Table 1). Two-way ANOVA revealed a significant main effect of stress on risk assessment in the LDT as well as all outcomes in the EPM test. Additionally, the main effect of genotype was significant for SI, distance and velocity in the EPM test, and nesting score. No significant interaction effect was observed on any of the behavioral measures.

**Fig. 2:**
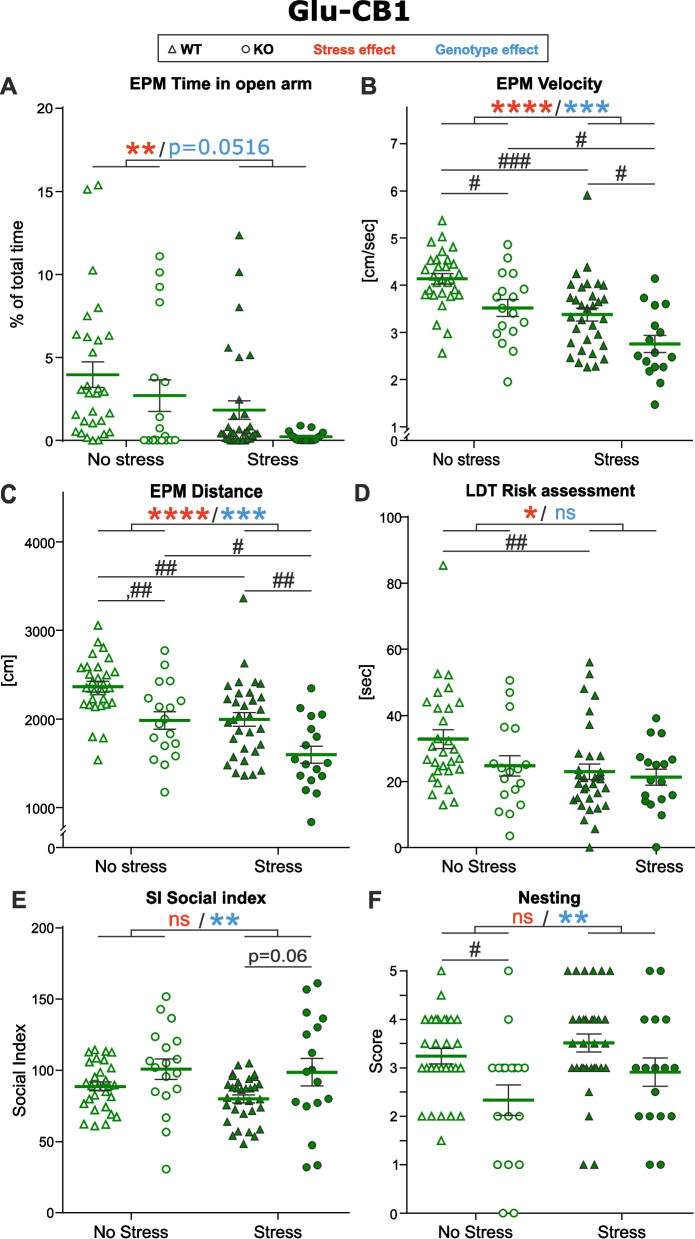
Glu-CB1-KO phenotype. **A-C **Elevated plus maze (EPM). **D **Light-dark test (LDT). **E** Social interaction test (SI). **F** Nesting test. The effect of experimental factors was evaluated with a two-way ANOVA including an interaction term followed by Tukey’s multiple comparisons test. Asterisks indicate the significance of one of main effects from the two-way ANOVA test: * *p*˂0.05, ** *p*˂0.01, *** p˂0.0005, **** *p*˂0.0001. Number signs indicate the significance of pairwise comparisons: # *p*˂0.05, ## *p*˂0.01, ### *p*˂0.0005. Data represented as mean ±SEM and individual values. WT No Stress/Stress *n*=29/32, KO No Stress/Stress *n*=18/17

Post-hoc pairwise comparisons for the effect of stress showed that stressed mice spent significantly less time than non-stressed ones in the open arms of the EPM (Fig. 2 A, *p* = 0.0020), indicating increased anxiety levels and validating the effect of CSD. Moreover, we observed a marginally significant (*p* = 0.0516) effect of genotype: stressed and non-stressed KO mice spent less time in the open arms compared to stressed and non-stressed WT mice. It is worth noting a tendency (*p* = 0.7784), where non-stressed KO mice showed behavior intermediate to that of non-stressed and stressed WT animals. This suggests a potential, albeit subtle, contribution of the genotype to baseline anxiety-like behavior in this paradigm.

Velocity and total distance traveled in EPM were also decreased in stressed groups (Fig. 2B, *p* < 0.0001; Fig. 2C, *p* < 0.0001 respectively), while there was also a significant decrease in movement in both stressed and non-stressed KO mice (Fig. 2B velocity, *p* = 0.0001; Fig. 2C distance, *p* < 0.0001). Post-hoc pairwise comparison of groups revealed that under basal conditions the KO mice moved significantly less and slower compared to WT. This effect is more prominent in stressed KO mice, having significantly reduced velocity and decreased distance moved than both non-stressed controls and stressed WT. Stress also had a negative effect on the mobility of WT, although to a lesser extent.

Furthermore, the stress procedure significantly impacted the total time of risk assessing behavior in the LDT (Fig. 2D, *p* = 0.0219), measured when the animal was detected in the entry zone of the LDT apparatus. Although stressed WT mice spent significantly less time assessing risks compared to non-stressed WT, the same trend was not observed in non-stressed vs. stressed KO. Total time spent in the light zone of the LDT was decreased in stressed groups; however, the effect was not significant (*p* = 0.0735, data not shown).

When comparing KO mice to the respective littermate WT controls in both stressed and non-stressed groups, KO spent more time interacting with the social target in the SI test (Fig. 2E, *p* = 0.0044). However, this effect was independent of stress (*p* = 0.2988). KO animals generally showed higher variability in the SI test. Stressed KO also interacted more with the social target than stressed WT, although this effect was only marginally significant (*p* = 0.06), potentially due to the abovementioned higher variability in the KO group.

Additionally, KO mice build significantly worse nests (Fig. 2F, *p* = 0.0014), but the effect of stress exposure was not significant (*p* = 0.0665). The pairwise comparison revealed that under baseline conditions, KO mice build significantly worse nests than WT, although the same trend was not confirmed after stress.

No stress, genotype or interaction effects were observed in the NOH (stress: *p* = 0.9255, genotype: *p* = 0.4134) and TST (stress: *p* = 0.2416, genotype: *p* = 0.1757).

In summary, mice lacking CB1 on cortical glutamatergic neurons under baseline conditions exhibit more anxious behavior, as compared to WT littermates, in the EPM and LDT and also worse performance in routinely performed tasks, as revealed by the nesting test. This behavior is further potentiated by CSD, as seen in EPM. Surprisingly, Glu-CB1-KO tend to interact more with the social target in the SI test, although there was high variability in response to the social cue among KO mice compared to WT.

### Effects of conditional knockout of NAPE-PLD, the main synthetizing enzyme of AEA, in cortical glutamatergic neurons on behavior after CSD stress

Nex-NAPE-PLD-KO mice are devoid of the NAPE-PLD enzyme in Nex-expressing neurons resulting in a decrease of AEA signaling in cortical glutamatergic neurons.

**Fig. 3:**
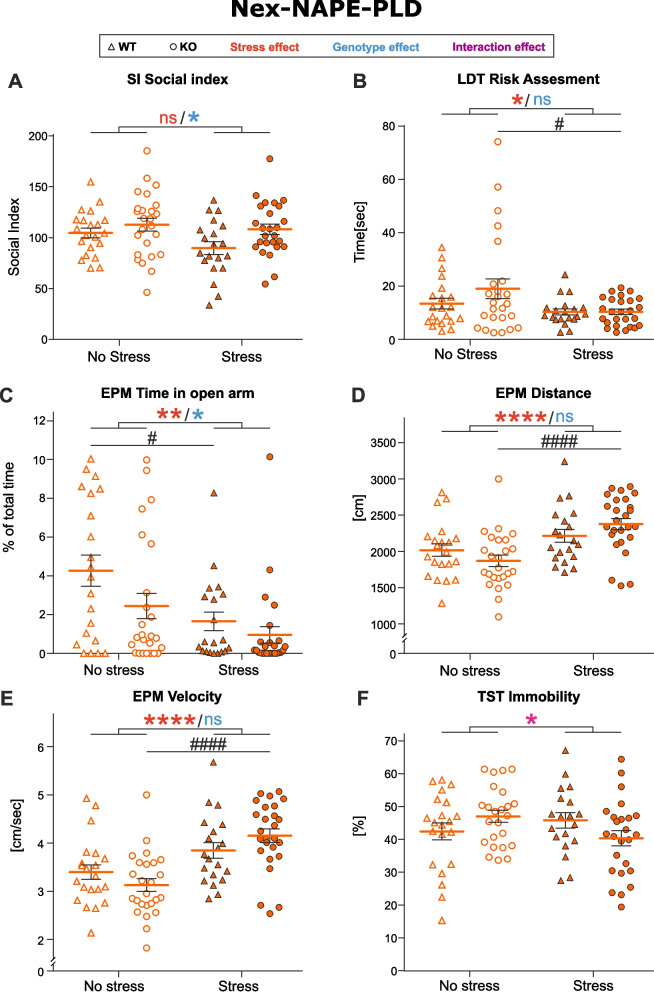
Nex-NAPE-PLD phenotype. **A** Social interaction (SI). **B **Light-dark test (LDT). **C-E** Elevated plus maze (EPM). **F** Tail suspension test (TST). The effect of experimental factors was evaluated with a two-way ANOVA including an interaction term followed by Tukey’s multiple comparisons test. Asterisks indicate the significance of one of main effects from the two-way ANOVA test: * *p*˂0.05, ** *p*˂0.01, **** *p*˂0.0001. Number signs indicate the significance of pairwise comparisons: # *p*˂0.05, #### *p*˂0.0001. Data represented as mean ±SEM and individual values. WT No Stress/Stress *n*=21/20, KO No Stress/Stress *n*=25/26

After exposing Nex-NAPE-PLD mice to CSD, we observed an increased preference of KO animals for social target in SI (Fig. 3 A, *p* = 0.0239), independently of stress (*p* = 0.0946). Increased anxiety-like behavior was observed in LDT and EPM; these and other findings are summarized in Supplementary Table 3 and described in detail below.

In particular, stressed and non-stressed KO mice showed a tendency to spend more time in the light compartment of the LDT compared to stressed and non-stressed WT mice, although the two-way ANOVA test did not reveal any significant differences of stress (*p* = 0.1678, data not shown) or genotype (*p* = 0.4096, data not shown). However, stressed animals spent less time in the entry zone of the LDT apparatus, indicating reduced risk assessment behavior (Fig. 3B, *p* = 0.0143). Moreover, pairwise comparisons revealed a significant decrease in risk assessment behavior in stressed KO mice as compared to non-stressed KO.

The EPM highlighted that the stressed mice of both genotypes spent less time in the open arm of the apparatus as a proxy of an anxiogenic effect of CSD (Fig. 3C, *p* = 0.0010). Moreover, pairwise comparisons revealed a significant decrease in time spent in the open arm of the EPM in stressed WT, compared to non-stressed WT. Additionally, anxiogenic behavior was exacerbated in both stressed and non-stressed KO mice (Fig. 3C, *p* = 0.0392).

Furthermore, both the total distance travelled and velocity in the EPM were significantly increased in stressed mice (Fig. 3D-E, *p* < 0.0001 for both distance and velocity); these parameters exclude any motor function deficits in the stressed groups, attributing reduction of the time spent in the open arm of the EPM to increased anxiety-like behavior.

However, there is a surprising marginally significant interaction effect of stress and genotype (distance *p* = 0.0630, velocity *p* = 0.0528). Non-stressed KO mice moved slightly slower and less, compared to non-stressed WT, whereas this effect was reversed in stressed groups: stressed KO mice move more and faster than stressed WT. A similar interaction effect (Fig. 3F, *p* = 0.0308) was observed in TST: non-stressed KO mice were immobile longer than non-stressed WT; whereas in the stress groups, stressed KO mice showed decreased immobility as a proxy of better coping behavior than stressed WT. The pairwise comparison did not reveal any significant group differences in the TST. Therefore, the antidepressant-like effect observed in TST in Nex-NAPE-PLD KO mice cannot be stated definitively.

No stress, genotype or interaction effects were observed in NOH (stress: *p* = 0.932, genotype: *p* = 0.904) and Nesting (stress: *p* = 0.384, genotype: *p* = 0.403).

To summarize, mice expected of having decreased levels of AEA in cortical glutamatergic neurons show increased social behavior under baseline conditions, as well as after CSD. On the other hand, Nex-NAPE-PLD-KO mice exhibit increased anxiety-like behavior in the EPM, in accordance with our previous results (Tevosian et al. 2023). Significant increase in traveled distance and velocity in the EPM in stressed KO, as compared to non-stressed KO, might indicate a slightly hyperactive behavior, partly explaining higher sociability in SI despite the anxiogenic phenotype in EPM.

### Effects of conditional knockout of FAAH, the main degradation enzyme of AEA, in cortical glutamatergic neurons on behavior after CSD stress

Nex-FAAH-KO mice are devoid of the FAAH enzyme in cortical glutamatergic neurons, leading to an increase of AEA signaling in these neurons. The behavioral test battery, performed after CSD, revealed an increase in anxiety-like behavior. These and other findings are summarized in Supplementary Table 3.

**Fig. 4:**
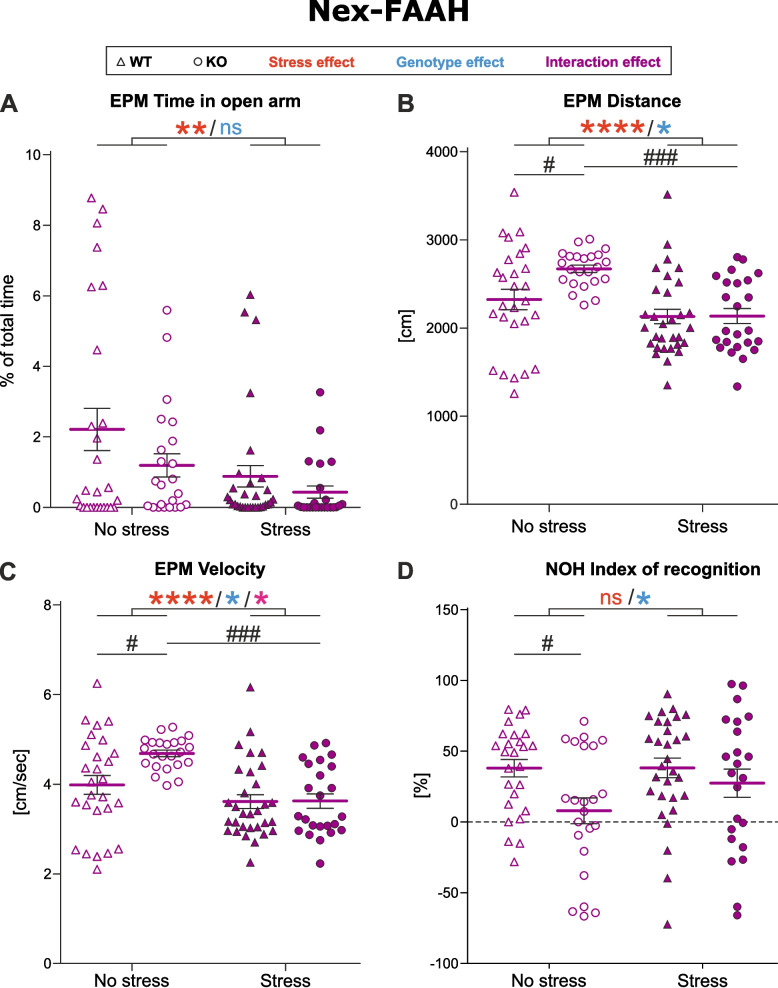
Nex-FAAH phenotype. **A-C** Elevated plus maze (EPM). **D** Novel object habituation (NOH). The effect of experimental factors was evaluated with a two-way ANOVA including an interaction term followed by Tukey’s multiple comparisons test. Asterisks indicate the significance of one of main effects from the two-way ANOVA test: * *p*˂0.05, ** *p*˂0.01, **** *p*˂0.0001. Number signs indicate the significance of pairwise comparisons: # *p*˂0.05, ### *p*˂0.0005. Data represented as mean ±SEM and individual values. WT No Stress/Stress *n*=27/31, KO No Stress/Stress *n*=23/24

In detail, we observed a strong anxiogenic effect of CSD in the EPM test. Stressed mice spent less time in the open arms of the EPM (Fig. 4A, *p* = 0.0097). This effect was more pronounced in KO mice, although the two-way ANOVA test revealed only a marginal effect of the genotype (*p* = 0.0674).

Interestingly, EPM also revealed an effect of CSD on the locomotion of Nex-FAAH mice. Although locomotion was reduced in both stressed groups (Fig. 4B, C, effect of stress, *p* < 0.0001 in velocity and distance), two-way ANOVA test also indicated an effect of genotype (Fig. 4B, C, distance, *p* = 0.0497; velocity, *p* = 0.0324) and a significant interaction (Fig. 4B, C, distance, *p* = 0.0549; velocity, *p* = 0.0381), pointing to a differentially directed response to stress between WT and KO. Tuckey’s multiple comparisons post-hoc test revealed slight hyperlocomotion in non-stressed KO mice compared to non-stressed WT: the velocity and distance moved of KO mice was increased (Fig. 4B, C, velocity: *p* = 0.0199, distance: *p* = 0.0379). Furthermore, stressed KO were comparable to stressed WT mice (velocity: p˃0.9999, distance: p˃0.9999) but significantly different from non-stressed KO (Fig. 4B, C, velocity: *p* = 0.0002, distance: *p* = 0.0005).

In contrast, there was no difference in time spent in the light compartment of LDT, as well as in time spent in the entry zone (risk assessment behavior), but the number of entrances to the entry zone was marginally decreased in stressed KO and WT (*p* = 0.0534, data not shown).

NOH revealed an impairment in short-term memory in KO mice (Fig. 4D, *p* = 0.0122), exploration inhibition was decreased in non-stressed KO mice (*p* = 0.0381) but restored to WT levels in stressed group (*p* = 0.7660).

No effect of either stress or genotype was observed in SI (stress: *p* = 0.2201, genotype: *p* = 0.7649), TST (stress: *p* = 0.9273, genotype: *p* = 0.5608) and Nesting (stress: *p* = 0.1893, genotype: *p* = 0.5101).

To summarize, Nex-FAAH-KO mice lacking FAAH in cortical glutamatergic neurons show a tendency for increased anxiety-like behavior after CSD. Interestingly, motor functions in KO animals seem to be affected at baseline conditions compared to WT controls, as these animals show hyperlocomotion in the EPM. Stressed animals, however, demonstrate locomotion comparable to stressed WT. Effects of stress on short-term memory of KO mice are similar, although additional behavior tests have to be performed to assess cognition and memory.

### Confirmation of changed levels of NAPE-PLD and FAAH by Western blot

**Fig. 5:**
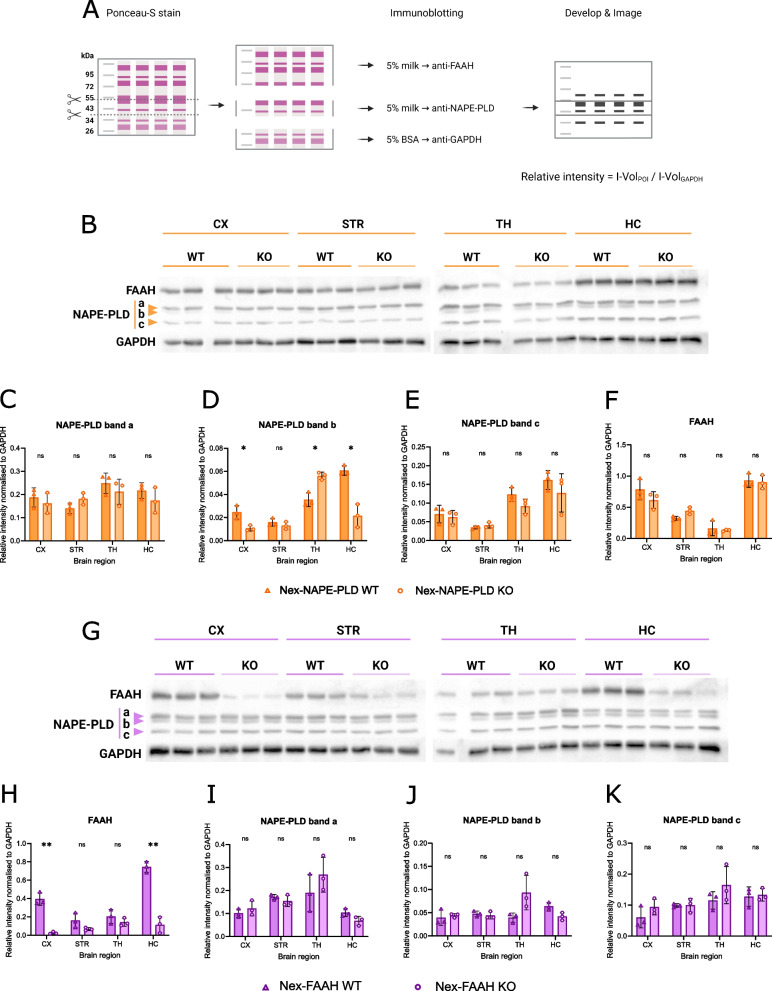
Western blot analysis of NAPE-PLD and FAAH in Nex-NAPE-PLD and Nex-FAAH mice. **A** Schematic representation of the experimental approach. The Intensity Volume (I-Vol) of the bands of interest (FAAH and NAPE-PLD) was divided by the I-Vol of the endogenous control band (GAPDH) within the same sample to generate the relative intensity; these values were plotted for each band of interest between genotypes and brain regions. **B** Western blots immunostained against FAAH, NAPE-PLD (bands a-c) and GAPDH. Quantification of NAPE-PLD (**C-E**) and FAAH (**F**) in cortex (CX), striatum (STR), thalamus (TH) and hippocampus (HC) of NEX-NAPE-PLD wild-type (WT, triangles) and knock-out (KO, circles). **G **Western blots immunostained against FAAH, NAPE-PLD (bands a-c) and GAPDH. Quantification of the FAAH (**H**) and NAPE-PLD (**I-K**) in cortex (CX), striatum (STR), thalamus (TH) and hippocampus (HC) of NEX-FAAH wild-type (WT, triangles) and knock-out (KO, circles). Asterisks indicate the significance of the multiple unpaired t-test with Holm-Šídák correction method: * *p*˂0.05, ** *p*˂0.01. Data represented as mean ±SD, *n*=3 (samples per genotype per mouse line)

To confirm the successful gene deletion in Nex-NAPE-PLD and Nex-FAAH mouse lines, we selected 2 cortical (cortex (CX) and hippocampus (HC)) and 2 subcortical (striatum (STR) and thalamus (TH)) brain regions and analyzed the content NAPE-PLD and FAAH by Western Blot. By employing Western blot membrane sectioning, we were able to quantify both proteins of interest from the same blot, reducing technical variability (see Fig. 5A). Notably, we detected 3 bands on the Western blots for the NAPE-PLD protein (Fig. 5B). Band c was at ca. 45 kDa and was the one indicated by the antibody provider. Bands a and b were at 54 and 50 kDa, respectively. Since the NAPE-PLD antibody is polyclonal, these findings are not concerning. The NAPE-PLD protein content was significantly reduced in the cortical regions of Nex-NAPE-PLD KO samples (Fig. 5D, CX *p* = 0.038, HC *p* = 0.013). There was a tendency to decreased levels of NAPE-PLD in TH (band a) and HC (bands a and c), albeit non-significant (Fig. 5C, E). We observed a drastic reduction of the FAAH protein content in cortical regions in Nex-FAAH KO samples (Fig. 5G-H, CX p = 0.002, HC p = 0.002). Notably, the FAAH content was higher in CX and HC, resulting in the strongest differences between Nex-FAAH WT and KO samples. The changes in STR and TH were less pronounced and not significant, as expected. The NAPE-PLD protein content was not changed significantly in selected brain regions of Nex-FAAH mice (Fig. 5I-K). These findings validate the genetic manipulation of both Nex-NAPE-PLD and Nex-FAAH mouse lines.

## Discussion

### Cell-type specific versus neuronal activity regulated recombination

Conditional gene deletion using Nex-Cre to ablate CB1, NAPE-PLD, or FAAH from cortical glutamatergic neurons from early stages of development did not yield a coherent phenotype in this study. The eCBs, as well as CB1 and CB2 receptors, play an essential role in neurodevelopment: neuronal proliferation and migration, differentiation, synaptogenesis, and myelinogenesis (Gaffuri et al. 2012; Basavarajappa et al. 2009). CB1 is highly expressed from early fetal stages (embryonic day 12.5 (E12.5)) in mice. The expression pattern is different from the adult brain: CB1 is highly expressed in glutamatergic projection neurons from E12.5 and is later downregulated from postnatal day 5 (P5) (Vitalis et al. 2008). Expression of Nex-Cre starts at E11.5, overlapping with the CB1 expression, specifically in glutamatergic neurons. NAPE-PLD expression starts at E10.5 and sharply peaks at P1. The expression of FAAH fluctuates during embryonic development, starting at E7.5 and reaching maximum levels at postnatal stages. AEA can be detected in the developing brain as early as E14.5 (Psychoyos et al. 2012). Therefore, affected neurodevelopment might be a factor influencing the behavior of mice, irrespective of the CSD stress. For instance, we observed decreased basal locomotion in EPM in Glu-CB1-KO and Nex-FAAH-KO mice, as well as an increase in basal social preference in SI test in Glu-CB1-KO and Nex-NAPE-PLD-KO mice.

Since it is impossible to disentangle the influence of neurodevelopmental alterations on the behavior of these mice from the impact of chronic stress, we suggest employing a time-specific KO of the elements of the eCB system. Rather than focusing only on glutamatergic neurons, one can make use of a recently developed TRAP system, allowing to genetically manipulate only a subset of neurons that were active during a selected timepoint (Tevosian et al. 2023).

### Limitation of short-term behavioral testing

When using congenic inbred mouse lines for biomedical and, in particular, behavioral research, it might be expected that the response to a stimulus or performance in a task will be rather homogeneous, particularly under highly standardized experimental conditions. However, high variability of response in behavioral tests between littermate cage-mates can be attributed to different factors such as the intrauterine position of the embryo that influences its hormonal milieu, maternal stress, continuing with postnatal stress, and social status (Lathe 2004). The effects that are sought for in contemporary neuroscience are much smaller and weaker than 50 years ago (Button et al. 2013). Therefore, careful consideration of the sample size and the power of the study has to be performed. Moreover, it is not advisable to perform multiple studies on small sample sizes and combine results (Rizzo and Silverman 2016). A higher-powered (large sample-sized) study, accounting for the individuality of a mouse, is more likely to detect subtle changes in mouse phenotype (Garner 2014). We observed high inter-individual variability in all performed behavior tests, e.g., animals displaying between 15% and 60% immobility time in the TST in Nex-NAPE-PLD-WT non-stressed controls. By employing large group sizes, we intended to overcome high baseline variability in responses and distill stress or genotype effects in this study.

Each behavioral test has its sources of variability, and only a subset of parameters is taken into account for the analysis (Hanell and Marklund 2014). By combining several behavioral tests targeting the same modality, e.g. EPM and LDT for anxiety-like behavior, we aimed at avoiding the chance of false-positive results (Garner 2014). Longitudinal home-cage observation, instead of acute behavioral tests, offers crucial advantages. The animal is not introduced to a novel anxiogenic environment and exhibits certain control over experimental conditions (Hager et al. 2014; Richter 2020). Additionally, the performance of an animal in a test can be monitored over multiple days or weeks, ensuring robust trait detection and behavior assessment during the active (dark) phase of mice. However, home cage behavior tracking is an emerging and developing field of behavioral neuroscience, with so far limited tools and solutions. In future studies, we plan to address the limitations of short-term testing by additionally employing home-cage longitudinal observations. The latter might help disentangle the effects of stress from the baseline differences in the non-stress groups.

### Categorization of mice into sub-groups

It is noteworthy to mention that no categorization of mice into resilient and susceptible sub-groups, based on their performance in the SI test after CSD stress, was performed in this study, contrary to common practice (Krishnan et al. 2007). In the original publication by Krishnan et al., a very high number of mice was used (ca. 300 mice per group), which justified the separation of the stressed group of animals into two sub-groups, based on their sociability score in the SI test, concomitant with anhedonia (assessed in the sucrose preference test). This approach was validated on behavioral (Krishnan et al. 2007), transcriptional (Bagot et al. 2016), electrophysiological (Hultman et al. 2018), and metabolic (Hamilton et al. 2020) levels, enriching the field of chronic stress and depression research with many valuable insights. However, these studies focused on extreme phenotypes, lying at the edge of the Gaussian distribution curve and driving the difference between groups. While this approach targets major differences, the behavior of the majority of the population is not taken into account.

Since no stress effect was observed in the SI in any of the mouse lines, the separation into subgroups, which would subsequently affect the analysis of all other behavior tests, was not justifiable in our eyes and therefore omitted.

### Female mice in chronic stress research

One of the limitations of the present study is the fact that only male subjects were used. The selected chronic stress model – social defeat – can be performed only on male mice, since it requires the territorial behavior for bouts of attack of the intruder by the resident. There are attempts to apply homologous stress on female mice by applying male odorants to females, which induces aggressive behavior in resident male mice (Harris et al. 2018), or by inducing aggressive behavior in males towards females using chemogenetic activation of the ventromedial hypothalamus (Takahashi et al. 2017). Unfortunately, these mice are far less common and studied. An introduction of a stress model, valid for both sexes, was proposed recently – the social instability stress (Yohn et al. 2019). Exposure of both male and female mice to novel cage-mates every 3 days over the course of 7 weeks prompts stress-induced behavioral changes and activation of the HPA axis. This novel method might help integrating female subjects into the field of chronic stress research, especially considering reported sexual dimorphism of the eCB system (Woodward et al. 2024).

## Conclusions

In conclusion, genetic manipulation of the endocannabinoid system in cortical glutamatergic neurons did not result in a pronounced phenotype after prolonged stress exposure. While we could confirm a robust anxiogenic effect of CSD in the EPM test in all tested lines, the genotype effect was not always present, besides the Nex-NAPE-PLD line. Interestingly, we have not observed a stress effect on the sociability of any of the mouse lines as identified in the SI test. Detected differences between the genotypes in the non-stressed groups, as observed in SI and Nesting in Glu-CB1 mice, SI in Nex-NAPE-PLD mice, and NOH in Nex-FAAH mice, points toward baseline differences that could mask or over-power the effect of stress.

Unlike Nex-FAAH-KO and Nex-NAPE-PLD-KO mice, the Glu-CB1-KO mice were previously assessed in behavior studies. However, Glu-CB1-KO mice were not characterized after a prolonged stress exposure, but rather under baseline or mildly aversive conditions (Rey et al. 2012; Jacob et al. 2009; Häring et al. 2011; Legler et al. 2015; Steiner et al. 2008). We could reproduce previously reported decreased locomotion (Dubreucq et al. 2012; Ruehle et al. 2013; Häring et al. 2011) and no effect of stress on the time spent in open arms of the EPM (Dubreucq et al. 2012). However, the sociability of the Glu-CB1-KO mice was increased after stress exposure, while non-stressed controls exhibited no differences between WT and KO animals, contrary to previous reports (Jacob et al. 2009; Häring et al. 2011). The disagreement might be attributed to a different execution of the SI test (previously used 2 chambers versus single-chamber apparatus used in the current study), as well as differences in data scoring (time of contact versus sociability index, respectively). However, some previous studies also report no differences in sociability of male mice (Terzian et al. 2014), supporting our findings.

We employed the previously not characterized mouse lines Nex-NAPE-PLD and Nex-FAAH in this study. Under baseline non-stress conditions, we observed an anxiogenic phenotype Nex-NAPE-PLD KO and hyperlocomotion in Nex-FAAH KO mice. Additionally, we confirmed the successful inactivation of NAPE-PLD and FAAH in cortical brain regions of respective KO mouse lines by Western Blot, validating the mouse lines.

Since the alterations are present from early development, compensatory mechanisms are possibly activated to counterbalance the lack of manipulated elements of the eCB system. The lack of synthesizing enzyme NAPE-PLD could be compensated by employing alternative synthesis pathways. Similar mechanisms could be activated to compensate for the lack of the degrading enzyme FAAH. Time-specific inactivation of CB1, NAPE-PLD, and FAAH could provide more insights (Tevosian et al. 2023; Monory et al. 2024), bypassing confounding effects of compensatory mechanisms during development.

## Supplementary Information

Supplementary Material 1.

## Data Availability

The datasets used and/or analyzed during the current study are available from the corresponding author on reasonable request.
