# Supplementary material for: Characterization of cell-type specific knockout of different elements of the endocannabinoid system in cortical glutamatergic neurons in the context of stress-induced behavioral phenotype
Source: J Cannabis Res. 2025 Nov 27;7:99. doi: 10.1186/s42238-025-00368-7 (PMC12667190; doi:10.1186/s42238-025-00368-7)
Supplement: Supplementary file 1 — Supplementary Material 1. [file 42238_2025_368_MOESM1_ESM.docx]

**SUPPLEMENTARY TABLES**

| **Test** | **Effect of stress**  **No stress vs. Stress** | **Significance** | **Effect of genotype**  **WT vs. KO** | **Significance** | **Interaction** |
| --- | --- | --- | --- | --- | --- |
| SI | Ø | Ø | ↑ | ****** | Ø |
| LDT Duration in light | Ø | Ø | Ø | Ø | Ø |
| LDT Risk assessment | ↓ | ***** | Ø | Ø | Ø |
| EPM Time in open arm | ↓ | ****** | Ø | Ø | Ø |
| EPM Distance | ↓ | ******** | ↓ | ******** | Ø |
| EPM Velocity | ↓ | ******** | ↓ | ******* | Ø |
| NOH | Ø | Ø | Ø | Ø | Ø |
| Nesting | Ø | Ø | ↓ | ****** | Ø |
| TST | Ø | Ø | Ø | Ø | Ø |

**Supplementary Table 1 Glu-CB1 KO.** WT No Stress/Stress n=29/32, KO No Stress/Stress n=18/17. Two-way ANOVA was used to test for genotype or stress effect, *p≤0.05, ** p≤0.01, ***p≤0.005, ****p≤0.001, Ø no effect, ↑ increased, ↓ decreased.

| **Test** | **Effect of stress**  **No stress vs. Stress** | **Significance** | **Effect of genotype**  **WT vs. KO** | **Significance** | **Interaction** |
| --- | --- | --- | --- | --- | --- |
| SI | ↓ | Ø | ↑ | ***** | Ø |
| LDT Duration in light | ↑ | Ø | Ø | Ø | Ø |
| LDT Risk assessment | ↓ | ***** | Ø | Ø | Ø |
| EPM Time in open arm | ↓ | ****** | ↓ | ***** | Ø |
| EPM Distance | ↑ | ******** | Ø | Ø | p=0.063 |
| EPM Velocity | ↑ | ******** | Ø | Ø | p=0.052 |
| NOH | Ø | Ø | Ø | Ø | Ø |
| Nesting | Ø | Ø | Ø | Ø | Ø |
| TST | Ø | Ø | Ø | Ø | ***** |

**Supplementary Table 2 Nex-NAPE-PLD.** WT NoStress/Stress n=21/20, KO NoStress/Stress n=25/26. Two-way ANOVA was used to test for genotype or stress effect, *p≤0.05, ** p≤0.01, ***p≤0.005, ****p≤0.001, Ø no effect, ↑ increased, ↓ decreased.

| **Test** | **Effect of stress**  **No stress vs. Stress** | **Significance** | **Effect of genotype**  **WT vs. KO** | **Significance** | **Interaction** |
| --- | --- | --- | --- | --- | --- |
| SI | Elongated distribution | Ø | Ø | Ø | Ø |
| LDT Duration in light | Ø | Ø | Ø | Ø | Ø |
| LDT Risk assessment | ↓ | Ø | Ø | Ø | Ø |
| EPM Time in open arm | ↓ | ****** | ↓ | p=0.067 | Ø |
| EPM Distance | ↓ | ******** | ↓ | ***** | p=0.054 |
| EPM Velocity | ↓ | ******** | ↓ | ***** | ***** |
| NOH | Ø | Ø | ↓ | ***** | Ø |
| Nesting | Ø | Ø | Ø | Ø | Ø |
| TST | Ø | Ø | Ø | Ø | Ø |

**Supplementary Table 3 Nex-FAAH.** WT NoStress/Stress n=27/31, KO NoStress/Stress n=23/24. Two-way ANOVA was used to test for genotype or stress effect, *p≤0.05, ** p≤0.01, ***p≤0.005, ****p≤0.001, Ø no effect, ↑ increased, ↓ decreased.
